# The Preventive Effects of *Asparagus officinalis* Extract on Sodium Selenite-Induced Cataractogenesis in Experimental Animal Models

**DOI:** 10.1155/2020/3708730

**Published:** 2020-11-23

**Authors:** Momammad Azadbakht, Mohammad Asghari, Kiumars Nowroozpoor Dailami, Ali Davoodi, Amirhossein Ahmadi

**Affiliations:** ^1^Department of Pharmacognosy and Biotechnology, Faculty of Pharmacy, Mazandaran University of Medical Sciences, Sari, Iran; ^2^Student Research Committee, Faculty of Pharmacy, Mazandaran University of Medical Sciences, Sari, Iran; ^3^Department of Ophthalmology, Bu-Ali Sina Teaching Hospital, Faculty of Pharmacy, Mazandaran University of Medical Sciences, Sari, Iran; ^4^Pharmaceutical Sciences Research Center, Faculty of Pharmacy, Mazandaran University of Medical Sciences, Sari, Iran; ^5^Hemoglobinopathy Research Institute, Mazandaran University of Medical Sciences, Sari, Iran

## Abstract

**Materials and Methods:**

Neonatal rats received a single dose of sodium selenite as an intraperitoneal injection (30 *μ*mol/kg) on day 10 postnatal to induce cataract. Animals were then posttreated with various oral solutions of *A. officinalis* extract at 200 mg/kg or 400 mg/kg once daily on days 10–16 postnatal. Cataract was evaluated by slit-lamp, and lens opacification was analyzed in each group 24 hours after the last treatment at day seven postadministration of the extracts or vehicle. The total protein concentration of lenses, glutathione reductase activity as the glutathione antioxidant capacity, and malondialdehyde content as a marker of lipid peroxidation were further assessed in removed rat lenses on day 30 postnatal.

**Results:**

All lenses in the healthy and control plant groups were clear. Sodium selenite significantly increased cataract grade (2.8 ± 0.2) when compared to the healthy group (*p* = 0.001). However, cataract grades were decreased considerably as 1.9 ± 0.72 and 1.5 ± 0.85 in groups that received 200 mg/kg and 400 mg/kg oral extract of *A. officinalis*, respectively. *A. officinalis* extract also restored all abnormalities of biochemical markers induced by sodium selenite.

**Conclusion:**

Our data suggest that *A. officinalis* could be a promising candidate as a safe alternative treatment in cataracts upon further clinical trials. This effect is probably associated with the antioxidant activity of *A. officinalis*.

## 1. Introduction

Cataract is a disease that the eye lens becomes progressively opaque, resulting in blurred vision, cloudiness, or the opacification of the lens of the human eye [1–3]. Cataract is responsible for 51% of the world's blindness, representing approximately 20 million people [2, 4]. Surgical replacement of the opacified lens with an artificial lens is currently the only way to cure vision loss. Although cataract surgery is now considered the most successful cure in terms of visual outcome, the cost, need for trained personnel, and postsurgical complications limit the worldwide availability and accessibility to this procedure [5]. Thus, the development of an alternative for surgical intervention to prevent or delay the onset of cataract is needed. The pathogenesis of cataracts is multifactorial and involves mechanisms that are not fully understood; oxidative stress is the foremost cause of cataract formation [6–9].

Oxidative stress causes an imbalance between the rate of oxidant production and the rate of detoxification, so the rate of oxidant production is significantly higher than that of detoxification using antioxidants [10]. With age, oxidants accumulate while antioxidant defenses gradually diminish, which is likely the most crucial mechanism in age-related cataract formation [11]. Since oxidative stress is implicated in cataract formation, the application of antioxidant agents, which may be capable of successfully penetrating the lens tissue, may counteract oxidation-related cataractogenesis. Much herbal medicine and natural products with antioxidant effects have been found to prevent sodium selenite-induced cataracts. *Echium amoenum* is an Iranian indigenous medicinal plant. In (2015) study, we showed that *E. amoenum* extract significantly had a protective effect on selenite-induced cataract in rat. This effect is probably associated with the antioxidant activity of this medicinal plant, resulted in inhibiting oxidative stress caused by cataract [12]. In another experiment, we assessed the preventive effects of *Origanum vulgare* extract, a potent antioxidant herbal medicine, on selenite-induced cataractogenesis. *O. vulgare* acted as a powerful therapeutic plant against selenite-induced cataract formation. The anticataract effects of *O. vulgare* extract could be based on the high amount of flavonoids and polyphenolic compounds, resulting in the strengthen endogenous antioxidant enzymes defenses system of the body, inhibition of oxidative stress conditions in cataract, and scavenging free radicals that caused lipid peroxidation by direct or indirect antioxidant mechanisms [13].

**Figure 1:**
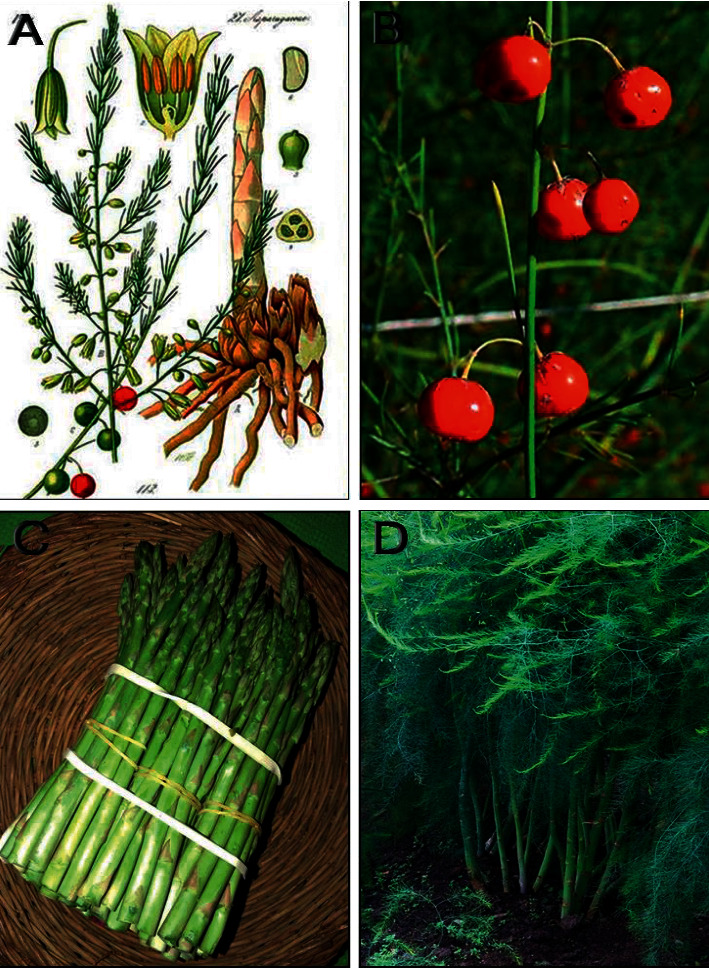
*Asparagus officinalis:* (a) Botanical illustrations. (b) Fruits. (c) A bundle of cultivated asparagus. (d) Aerial parts.

*Asparagus officinalis* L. is known as an anticataract plant in the canon of medicine in Persian Medicine and traditional medical books [14]. *A. officinalis* is one of the Asparagaceae family plants; it is a perennial and monocotyledonous plant that is grown for its edible young stems. Young shoots are harvested in spring and consumed as a seasonal vegetable (Figure 1). Asparagus extracts have been traditionally used as diuretics, laxatives, and antitussives. Recent studies have shown that young shoots have antioxidant and antitumor effects on humans [15]. Young shoots of *A. officinalis* contain specific amounts of phytosterols, such as *β*-phytosterol, which reduce blood cholesterol levels. Asparagus is primarily a rich source of flavonoids, including rutin and quercetin, and it is a source of vitamin B_6_, zinc, beta-carotene, vitamin C, vitamin E, vitamin K, thiamin, riboflavin, niacin, folic acid, phosphorus, copper, manganese, and selenium. The asparagus plant is rich of asparagine amino acid [15–18]. Thus, it is hypothesized that *A. officinalis* with its antioxidant compounds and excellent free radical scavenging may be having a protective action on oxidative stress conditions in selenite-induced cataractogenesis.

In the current study, we evaluated the effect of *A. officinalis* against selenite-induced experimental cataract in the rat model. Cataract was measured by slit-lamp, and lens opacification was analyzed in each group on day seven after selenite administration. The total protein concentration of lenses, glutathione reductase (GR) activity as the glutathione antioxidant capacity, and MDA as a marker of lipid peroxidation were further assessed in removed rat lenses.

## 2. Materials and Methods

### 2.1. Drugs and Chemicals

Sodium selenite (Merck Company, Germany, 99.9% pure) was used to induce cataracts in rats. Then, 1% tropicamide ophthalmic solution and 2.5% phenylephrine were used to dilate the pupil of the eye in preparation for the slit-lamp examination. Ketamine 10% injection vial, xylazine 2% injection vial (Alfasan, Netherlands), sodium chloride 0.9%, and Liposic® ophthalmic gel (Dr. Gerhard Mann Chem.-Pharm, Germany) were used.

### 2.2. Preparation of *A. officinalis* Extract

*A. officinalis* was collected from Sari Asparagus gardens, Sari, Iran. Young shoots of the plant were identified by systematic plant specialist Dr. Masoud Azadbakht, the Mazandaran University of Medical Sciences, Sari, Iran. To obtain the hydroalcoholic extract of *A. officinalis*, we needed to dry the plant. First, we cut the young asparagus shoots into smaller pieces and dried them in an oven at 42°C for 48 hours. The dried parts of the plant were then crushed until 200 mesh using a blender. Next, 500 g of dried young shoots of the plant was soaked through the maceration method, which uses 70% hydroalcoholic extract comprising 30% distilled water and 70% ethanol. The hydroalcoholic extract was drained three times and left at room temperature for three days. The extract was then filtrated and kept in light-proof head-tight bottles in a fridge. After that, the hydroalcoholic solvent was evaporated using a rotary evaporator at 40°C and then dried using a freeze-dryer.

### 2.3. Determination of Total Phenol Content

The total phenol content of the spice extract was determined using the Folin–Ciocalteu method [19]. This method is based on phosphotungstate-phosphomolybdate reduction in an alkaline medium. In this method, we followed the methods of Tankeu et al. 2016 [20]. 200 *μ*L of 1 mg/ml of samples was first introduced to different test tubes. Then, 800 *μ*L of 10-fold diluted Folin reagent and 2000 *μ*L of sodium carbonate solution (7.5%) were added. After stirring for 5 minutes, the mixture was kept away from light for 2 h, and the absorbance was measured using a spectrophotometer at 765 nm. The phenolic content was determined from a quercetin standard curve. A concentration range from 0 mg/mL to 0.3 mg/mL of quercetin was prepared to determine the total polyphenol content expressed in mg equivalents of quercetin/g of extract.

### 2.4. Determination of Total Flavonoid Content

The total flavonoid content was determined using the following method [21]. First, 100 *μ*L of the extract was added to 300 *μ*L of distilled water and 30 *μ*L of NaNO_2_ (5%) and then incubated for 5 minutes at 25°C. Next, 30 *μ*L of AlCl_3_ (10%) was added to the solution. After another 5-minute waiting time, the reaction mixture was treated with 200 *μ*L of 1 mM NaOH, and the reaction mixture was diluted to 1000 *μ*L with distilled water. Quercetin served to draw the standard calibration curve in the range of 0–0.3 mg/mL, and the absorbance was measured at 510 nm. The results were expressed as mg quercetin equivalents/g of dried extract.

### 2.5. Animals

Sixty white neonatal Wistar rats weighing 13–16 g and their mothers were purchased from the animal lab of the Mazandaran University of Medical Science. They were kept in animal housing at the animal facility of the Faculty of Pharmacy and maintained under a controlled 12 hr light/dark cycle and temperature (24 ± 1°C) in individual polyacrylic cages. The Institutional Review Board and the Research Committee of the University approved the study protocols. All experiments performed during the studies conformed to accepted principles for laboratory animal use and care (1986: 86/609).

### 2.6. Experimental Treatment

For the experiment, animals were divided into five groups (groups 1–5, *n* = 5 for each group) as follows: newborn rats received a single intraperitoneal (i.p) injection of NaCl 0.9% at day 10 postnatal with no further treatment as the control healthy group. A single dose of 30 *μ*mol/kg sodium selenite dissolved in NaCl 0.9% at day 10 postnatal was intraperitoneally injected to induce cataract. Animals were then posttreated with various oral solutions of *A. officinalis* extract at 200 mg/kg or 400 mg/kg once daily on days 10–16 postnatal. Besides, rats in one group received an oral solution of *A. officinalis* (400 mg/kg) on days 10–16 postnatal as the control plant group. No more than 0.5 ml of sodium selenite solution was administered to each rat. The observations of lens opacification were performed on day seven after sodium selenite administration by slit-lamp. All neonatal rats were anesthetized on day 30 postnatal by intraperitoneal injection of ketamine (80 mg/kg) and xylazine (15 mg/kg). Lenses were removed immediately after euthanasia and promptly placed on dry ice. Samples were stored at a temperature of −80°C for further analysis.

### 2.7. Cataract Grades

Cataracts were graded according to these scales; grade 0, clear lens; grade 1, a lens with slight opacity; grade 2, a lens with partial nuclear opacity; and grade 3, a lens with dense nuclear opacity.

### 2.8. Determination of Total Lens Protein

The lenses of each rat homogenized with the glass-glass homogenizer. The total protein concentration of homogenized lenses, which were obtained by enucleating the eyes of rats, was determined through the use of the bicinchoninic acid (BCA) protein assay kit (Pierce, Rockford, IL. USA). A total of 50 *μ*L of lens homogenate, standard bovine serum albumin (BSA), and working reagent (constituted as per the manufacturer's directive) were pipetted into a 96-well microplate. Absorbances were measured at 562 nm using a URIT-660 microplate reader (URIT Medical Electronic Co., Ltd, Guangxi, China). Each determination was in triplicate.

### 2.9. Determination of Glutathione Reductase (GR) Activity

We used the previously described Ellman method to determine the glutathione antioxidant capacity of plant extracts [22]. An aliquot of PBS (580 *μ*L), 200 *μ*L of extract, and 200 *μ*L of each homogenate lenses and 20 *μ*L of inducing solution was introduced in different test tubes. The tubes were then incubated at 37°C for one hour. Finally, the test solutions, 20 *μ*L and 3000 *μ*L, of Ellman reagent (phosphate buffer 0.1 M; pH 6.5; 2,2-dithiol-5,5′-dibenzoïc acid) were introduced into new test tubes. Glutathione concentrations were expressed in micromoles/L and determined.

### 2.10. Determination of Lipid Peroxidation

We evaluated the capacity of the extract to inhibit the lipid peroxidation process in lenses according to the following method [23]. In brief, 580 *μ*L of phosphate buffer (0.1 M; pH 7.4), 200 *μ*L of asparagus extract, and 200 *μ*L of each homogenate lens were introduced into different test tubes. Then, lipid peroxidation was initiated by adding 20 *μ*L of oxidizing solution (0.1 M HCl, FeCl_3_ 200 mM, 400 mM NTA, and 200 mM H_2_O_2_) in the mixture. The solutions were then placed in a waterbath at 37°C for 1 h. After incubation, 100 *μ*L of this mixture was pipetted and put in new test tubes, to which 1000 *μ*L of malondialdehyde (MDA) reagent, TCA (10%), and 1 ml of TBA (0.67%) were added to terminate the intended reaction. After this, the tubes were heated again at 100°C for 20 min and transferred to an ice bath to be cooled and centrifuged at 3000 rpm for 5 min. The optical density was measured at 535 nm, and the concentration of MDA was determined.

### 2.11. Statistical Analysis

The data are presented as the mean ± SD. Differences between group means were estimated using a one-way analysis of variance followed by Tukey's HSD test. A *p* value of less than 0.05 was considered to be significant.

## 3. Result

### 3.1. Total Polyphenols and Flavonoids Content

The mean total phenolics content of *A. officinalis* young shoots extract as mg gallic acid (GA) equivalent/g of dried extract was 283 ± 7.5 (GA) in mg/g. The mean total flavonoids content of *A. officinalis* young shoots extract as mg quercetin (QE) equivalent/g of dried extract was 14.93 ± 1.41 (QE) mg/g.

### 3.2. Morphological Examination of the Eye Lens

**Figure 2:**
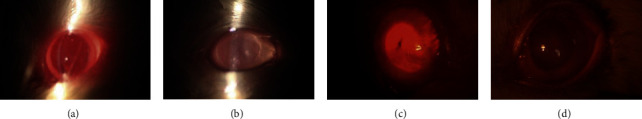
Grades 0–3 of lens opacity the eyes of rats checked by slit-lamp examination on day 17 postnatal. (a) Grade 0. (b) Grade 1. (c) Grade 2. (d) Grade 3.

**Figure 3:**
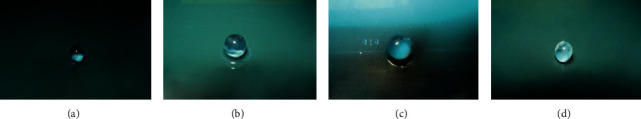
Grades 0–3 of lens opacity after enucleating the eyes of rats on day 30 postnatal. (a) Grade 0. (b) Grade 1. (c) Grade 2. (d) Grade 3.

Table 1 shows the results of the morphological analysis of rat eyes by slit-lamp on the 17^th^ day postnatal. The number of lenses of each group was sorted by the degree of opacity in four grades (grade 0–3). Cataract grades were 0 in normal, and the control plant group received only normal saline and an oral solution of *A. officinalis*, respectively. The mean grade of cataract in animals that received selenite was dramatically increased (2.8 ± 0.2) compared to healthy and control plant groups. In contrast, cataract grades were significantly decreased to 1.9 ± 0.72 and 1.5 ± 0.85 in groups treated by 200 mg/kg and 400 mg/kg oral extract of *A. officinalis*, respectively. Also, pictures of cataract grading of the lenses (with slit-lamp apparatus) on 17^th^ day postnatal and pictures of enucleated lenses after surgery on day 30 are shown in Figures 2 and 3, respectively. All lenses in normal and control plant groups were clear. In the single dose selenite injection group, most lenses had nuclear cataracts with high-density grade 3, and some of them showed grade 2 nuclear cataracts. In 200 mg/kg of the *A. officinalis* group, cataract grades were between 1 and 2, and in 400 mg/kg of the *A. officinalis* group, most of the lenses showed cataract with grade 1. Furthermore, the morphological reexamination of lenses on day 30 of lens removal surgery (Figure 3) confirmed the results of the initial examination of lenses (day 17), which is shown in Figure 2.

### 3.3. Biochemical Examination of the Eye Lens

**Figure 4:**
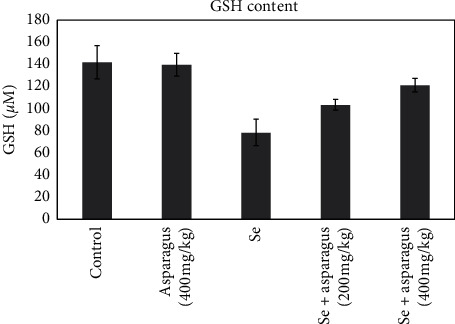
The amounts of lens GSH are represented as the mean ± SD of each group. ^###^*p* < 0.001, control sample compared with animals treated with a single dose of Se solution with a dose of 30 *μ*mol/kg. ^*∗*^*p* < 0.05, Se-injected mice as compared with oral-administrated mice with *Asparagus* (200 mg/kg and 400 mg/kg) after Se injection. ^*∗∗∗*^*p* < 0.001, Se-injected mice as compared with oral-administrated mice with only *Asparagus* (400 mg/kg). Asparagus, *Asparagus officinalis*. Se, sodium selenite.

The mean GSH level in the healthy group and control plant group was 141 ± 15.1 and 139.6 ± 10.3, respectively. The mean GSH level significantly decreased in the selenite group (78.6 ± 12). However, the GSH level was restored considerably as 103 ± 56 and 121.3 ± 6.11 in pretreated groups by 200 mg/kg and 400 mg/kg oral extract of *A. officinalis*, respectively, compared with the selenite group (Figure 4).

**Figure 5:**
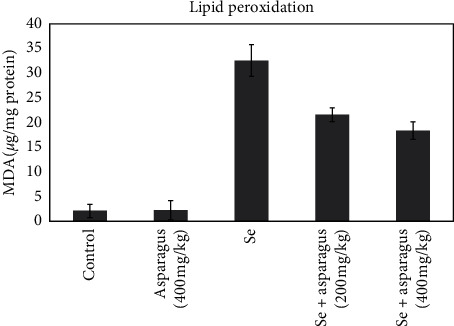
Lipid peroxidation activity presented as the amount of MDA in lenses is represented as the mean ± SD of each group of mice. ^###^*p* < 0.001, control sample compared with animals treated with a single dose of Se solution with a dose of 30 *μ*mol/kg. ^*∗*^*p* < 0.05, Se-injected mice compared with oral As-administrated mice (200 mg/kg and 400 mg/kg) after Se injection. ^*∗∗∗*^*p* < 0.001, Se-injected mice compared with oral As-administrated mice (400 mg/kg). As, *Asparagus officinalis*. Se, sodium selenite.

The mean MDA level as a lipid peroxidation marker in the healthy group and control plant group was 2.11 ± 2.352 and 2.28 ± 1.942, respectively. The mean MDA levels increased significantly in the selenite group as 32.57 ± 4.215, and the 200 mg/kg and 400 mg/kg of *A. officinalis* groups decreased to 18.37 ± 2.764 and 21.6 ± 2.391, respectively (Figure 5).

**Figure 6:**
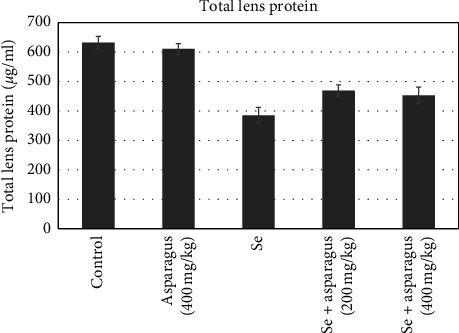
Total protein amounts of lenses are represented as the mean ± SD of each group. ^###^*p* < 0.001, control sample compared with animals treated with a single dose of Se solution with a dose of 30 *μ*mol/kg. ^*∗*^*p* < 0.05, Se-injected mice compared with oral As-administrated mice (200 mg/kg and 400 mg/kg) after Se injection. ^*∗∗∗*^*p* < 0.001, Se-injected mice compared with oral As-administrated mice (400 mg/kg). As, *Asparagus officinalis*. Se, sodium selenite.

The mean total protein content in the healthy group and control plant group was 631.7 ± 21.7 and 610.3 ± 18.4, respectively. The mean total protein content decreased significantly in the selenite group as 385.1 ± 26.9, and the 200 mg/kg and 400 mg/kg of *A. officinalis* groups increased to 452.8 ± 28.1 and 469.3 ± 19.5, respectively (Figure 6).

## 4. Discussion

We investigated the protective effect of *A. officinalis* young shoots extract against selenite-induced cataract in rat lenses for the first time. Evidence from previous studies suggests that reactive oxygen species and oxidative damage are involved in the development of cataracts [24, 25], a multifactorial disease. Oxidative stress in the lens is considered to be the most critical factor in the formation of cataracts [26]. Lens transparency is dependent on the maintenance of redox balance, which is, in part, maintained by its high GSH content [23]. Besides, studies have shown that lipid peroxidation has been associated with the formation of cataracts. Polyunsaturated fatty acids are susceptible to free radical attack, initiating a chain reaction that terminates in the creation of stable byproducts, such as MDA, which are used as markers of lipid peroxidation [27]. Therefore, as an alternative method to prevent or treat cataracts, antioxidant agents can be used to reduce lens oxidative stress.

In our study, lenses in the sodium selenite group had increased levels of MDA when compared to control group lenses, indicating increased lipid peroxidation in the sodium selenite group. This observation is the same as in our and other previous studies, which reported increased lipid peroxidation upon treatment with sodium selenite [12, 27, 28]. *A. officinalis* young shoots extract increased the amount of GSH. They also can decrease the lipid peroxidation induced by sodium selenite by providing adequate amounts of GSH, which is a substrate of the glutathione peroxidase-catalyzed reduction of hydroperoxides. In our study, there was no apparent difference between 200 mg/kg and 400 mg/kg doses of *A. officinalis* extract, either in biochemical results of antioxidant activity reduction or physical examination of lenses.

Selenite cataract is common in the rodent models used in the rapid screening potentiality of anticataract agents. This method was first described by Ostadalva et al. in 1987. After they receive a subcutaneous injection of 20–30 nmol/kg of sodium selenite by body weight, cataracts begin to form after 3-4 days in the lens of young rat eyes and are completed in a week [29]. Sodium selenite injection induces cataracts in young rats through various mechanisms, including calpain-induced hydrolysis and the precipitation of lenticular proteins [25]. Oxidative damage caused by selenite may involve the oxidation of critical sulfhydryl groups on calcium ATPase or ion channels [30]. Previous studies have shown that antioxidant agents can protect rat lenses from experimental selenite-induced cataract [12, 13].

*Asparagus officinalis* L., a well-known healthy vegetable, which was named as “the king of vegetables” for its abundant bioactive compounds, now is widely consumed all over the world. Bioactive components, such as flavonoid, lignan, and steroidal saponin, were found in this plant. In pharmacological studies, the extract from asparagus was shown to have several biological activities, such as antioxidant activity. Flavonoids and phenolics compounds were dominantly contributed to the antioxidant activity of the extract from asparagus residues [18]. *A. officinalis* polysaccharide is another bioactive component in asparagus. Like other plant polysaccharides, *A. officinalis* polysaccharide possesses a wide range of pharmacological properties, such as a significant function of scavenging hydroxyl radical [31]. Green asparagus polysaccharides could dramatically scavenge DPPH, hydroxyl, and superoxide anion free radicals and inhibit erythrocyte hemolysis and mitochondrial liver swelling. Green asparagus polysaccharides have good antioxidant effects in vitro [32]. In another investigation, aqueous extracts of green asparagus also exhibited radical scavenging, chelating activities, and protected liposome against oxidative damage. The high-performance liquid chromatography analysis indicated that polyphenolic components such as rutin, quercetin, kaempferol, and isorhamnetin were present in the aqueous extracts of green asparagus [33]. It is also known that caffeic and ferulic acids are relatively potent antioxidants. These acids were found in asparagus. The potent antioxidant activity of asparagus extract demonstrated a linear relationship with their flavonoid content [34]. Our data show that oxidative stress plays a role in cataract formation. The data support our hypothesis that *A. officinalis* extracts at 200 mg/kg and 400 mg/kg doses protect lenses by increasing GSH content, reducing lipid peroxidation, and restoring enzyme activity.

## 5. Conclusion

Our present biochemical and morphological results demonstrate that *A. officinal* extract inhibits the formation of Se-induced cataract, probably through its potent free radical scavenging and antioxidant properties. Thus, *A. officinal* extract is suggested to be used as an anticataract agent, although such therapeutic modality requires further clinical trials before it applies to patients. This present study and any future ones may eventually help prevent cataract formation in high-risk populations and treat early-stage cataracts without the need for surgical intervention that led to a decrease in the socioeconomic burden of blindness worldwide.

## Figures and Tables

**Table 1:** Results of rat eye examinations by slit-lamp on day 17 postpartum; grades 0–3 depend on lens opacification degree.

Groups number	Groups	Grades				
		Grade 0	Grade 1	Grade 2	Grade 3	%^*∗*^
Group 1	Normal saline	12	0	0	0	0
Group 2	*A. officinalis* (400 mg/kg)	12	0	0	0	0
Group 3	Sodium selenite	0	0	2	10	94.4
Group 4	Sodium selenite + *A. officinalis* (200 mg/kg)	0	4	5	3	63.8
Group 5	Sodium selenite + *A. officinalis* (400 mg/kg)	0	5	4	2	52.7

^*∗*^The percentage grade of each group is calculated based on the point value of each category. Grade% = ((total number of grade 0 × 0) + (total number of grade 1 × 1) + (total number of grade 2 × 2) + (total number of grade 3 × 3))/36 × 100.

## Data Availability

The data used to support this study are available from the corresponding author upon request.
